# Exogenous 6-Benzyladenine Improves Waterlogging Tolerance in Maize Seedlings by Mitigating Oxidative Stress and Upregulating the Ascorbate-Glutathione Cycle

**DOI:** 10.3389/fpls.2021.680376

**Published:** 2021-09-03

**Authors:** Ji Wang, Daye Wang, Min Zhu, Fenghai Li

**Affiliations:** College of Agronomy, Specialty Corn Institute, Shenyang Agricultural University, Shenyang, China

**Keywords:** AsA-GSH cycle, oxidative stress, waterlogging stress, waxy corn, 6-benzyladenine

## Abstract

The synthetic cytokinin 6-benzyladenine (6-BA) regulates plant growth and prevents the negative consequences of various forms of abiotic stress, including waterlogging in crop plants. The present study aimed to investigate the effects of exogenous 6-BA on the growth, oxidative stress, and ascorbate-glutathione (AsA-GSH) cycle system in the inbred SY-MY13 (waterlogging-resistant) and SY-XT1 (waterlogging-sensitive) seedlings of waxy corn in conditions of waterlogging stress. The results demonstrated that waterlogging stress causes chlorosis and necrosis in waxy corn leaves, inhibiting growth and leading to the accumulation of reactive oxygen species (ROS), which induces oxidative stress and, in turn, reduces membrane lipid peroxidation and the disruption of membrane homeostasis. This is specifically manifested in the increased concentrations of superoxide anion radicals (${\text{O}}_{2}^{-}$), hydrogen peroxide (H_2_O_2_), and malondialdehyde (MDA), in addition to increased relative electrical conductivity (REC%) values. The SY-MY13 strain exhibited growth superior to that of SY-XT1 when waterlogged due to its excellent waterlogging resistance. Thus, exogenous 6-BA was found to be effective in enhancing the growth of plants stressed by waterlogging in terms of the weight of the shoots and roots, shoot height, and leaf area. In addition to this, exogenous 6-BA also reduced the accumulation of ${\text{O}}_{2}^{-}$, H_2_O_2_, and MDA, increased ascorbate peroxidase (APX), glutathione reductase (GR), dehydroascorbate reductase (DHAR), and monodehydroascorbate reductase (MDHAR) activity, and enhanced ascorbic acid (AsA), and reduced glutathione (GSH) concentration through the regulation of the efficiency of the AsA-GSH cycle system in maize plants. Hence, the application of exogenous 6-BA can alleviate waterlogging-induced damage and improve waterlogging tolerance in waxy corn via the activation of the AsA-GSH cycle system and the elimination of ROS.

## Introduction

Climate change has compounded global environmental risks, affecting crop growth and jeopardizing food production and economic growth worldwide (Li et al., 2017; Tiwari et al., 2017; Maraci et al., 2018). Of the heightened environmental risks, waterlogging has already been identified as among the most critical abiotic stresses affecting crop productivity (Wright et al., 2017; Fukao et al., 2019). Waterlogging stress is defined as the saturation of the soil around the roots of crops with water, which creates a low-oxygen (hypoxic) environment because of the limited diffusion of gas in water (Xu et al., 2016; Ren et al., 2017).

Waterlogging stress modifies the balance between the endogenous production and neutralization of reactive oxygen species (ROS), such as superoxide anion radicals (${\text{O}}_{2}^{-}$), hydrogen peroxide (H_2_O_2_), etc., causing the accumulation of ROS that leads to oxidative stress in plants (Posso et al., 2018; Anee et al., 2019; Begum et al., 2019; Malhi et al., 2019; Park and Ju, 2019). Xiao et al. (2020) concluded that the exposure of peach seedlings to waterlogging induced the accumulation of intracellular reactive ROS, in turn, causing apoptosis and impeding their growth and development. Lou et al. (2017) observed that excess ROS increased lipid peroxidation, leading to the dysregulation of several physiological mechanisms in pakchoi. In addition, excess ROS also destroys physiological membrane activity, causing cell swelling and rupture (Zhang et al., 2007; Geetika et al., 2016; Sarwar et al., 2019).

Plants minimize the accumulation of ROS *via* complex and highly efficient enzymatic and non-enzymatic antioxidants (Ahmad et al., 2010, 2019; Kohli et al., 2019). Specifically, the ascorbate-glutathione (AsA-GSH) cycle has been reported to reduce the negative impact of ROS (Jung et al., 2020; Tan et al., 2020; Wang M. et al., 2020). The AsA-GSH cycle consists of the enzymes ascorbate peroxidase (APX), glutathione reductase (GR), monodehydroascorbate reductase (MDHAR), and dehydroascorbate reductase (DHAR), and the non-enzymatic molecules ascorbic acid (AsA), dehydroascorbic acid (DHA), reduced glutathione (GSH), and oxidized glutathione (GSSG) (Ahmad et al., 2010, 2019; Kohli et al., 2019). After preferentially donating an electron to APX, AsA becomes oxidized to monodehydroascorbate (MDHA) or DHA, while APX reduces H_2_O_2_ to H_2_O. MDHA is subsequently reduced to AsA by MDHAR, and DHA is reduced to AsA by GSH-dependent DHAR (Ahmad et al., 2010, 2019; Kohli et al., 2019). In a parallel reaction, GSH becomes oxidized by DHAR to generate GSSG, which is reduced back to GSH by GR (Foyer and Noctor, 2011; Kuo et al., 2020; Yildiztugay et al., 2020). Furthermore, the AsA-GSH cycle can regulate oxidative damage caused by environmental stresses, such as chromium stress in kenaf cultivars (Niu et al., 2018), waterlogging stress in wheat (Wang X. et al., 2016), drought stress in maize (Guo et al., 2020), and saline stress in rice (Islam et al., 2017). Thus, plants reduce the negative consequences of waterlogging stress through the up- or down-regulation of enzymatic and non-enzymatic antioxidants. Such studies have contributed to our understanding that the AsA-GSH cycle regulates the physiological response of plants to stress (Alam et al., 2019; Ahanger et al., 2020; Ahmad et al., 2020; Jan et al., 2020; Kaya et al., 2020).

Nevertheless, waterlogging stress results in several pernicious consequences that require sustainable and effective measures. One such intervention for alleviating waterlogging stress is supplementation with a plant growth regulator (PGR). PGR are often used to increase plant productivity, tolerance to stress, and self-repair qualitatively similar to the physiological and biological effects of phytohormones (Ali et al., 2015; Neill et al., 2019). Studies have demonstrated that PGRs can significantly increase the efficiency of the AsA-GSH cycle by removing the oxidative damage caused by the accumulation of ROS induced by environmental stress. Examples include heavy metal stress (Khan et al., 2019), salt stress (Alam et al., 2019), drought stress (Wang H. H. et al., 2016), and heat stress (Li Z., et al., 2016). Cell division is the most fundamental process of any system of biotic growth and development and core-to-tissue growth in all organisms (Alim et al., 2012; Meng et al., 2020). Cytokinins are central regulators of plant growth and development, playing important regulatory functions in physiological processes, including cell division, apical dominance, plant growth, photomorphogenesis, nutrient translocation, and leaf senescence (Paul et al., 2016; Wang Y. et al., 2016; Jan et al., 2019). In addition, cytokinins are also implicated in the regulation of plant immune signaling networks, suggesting that the growth and defense of plants are intimately connected (Van der Does et al., 2013; Sorensen et al., 2018). 6-benzyladenine (6-BA) is in a class of synthetic cytokinin PGRs that can significantly increase levels of endogenous cytokinins in crop plants, which otherwise decrease significantly when experiencing environmental stress (Hu et al., 2020; Prerostova et al., 2020). Thus, supplemental 6-BA allows plants to overcome the negative effects of various types of abiotic stress. Treatment with 6-BA has been shown to enhance photochemical efficiency, increase growth, and reduce Na^+^ accumulation in ryegrass in a saline environment; thus effectively preventing the inhibition of growth in plants experiencing salt stress (Ji et al., 2019). The exogenous application of 6-BA could significantly reduce toxicity caused by cadmium (Cd) and uranium (U) on rapeseed, subsequently reducing malondialdehyde (MDA) and H_2_O_2_ levels and increasing antioxidant levels (Chen et al., 2020). The addition of 6-BA also suppressed heat-induced leaf senescence and oxidative damage in ryegrass (Zhang et al., 2019) and enhanced drought stress tolerance in sweet potato plants (Li et al., 2020). Spraying wheat plants with 6-BA prior to becoming waterlogged has also been found to decrease MDA levels in leaves, reduce plant senescence, and facilitate the production of dry matter (Wang X. Y. et al., 2020). It has also been reported that exogenous 6-BA significantly increased the activity of the leaf defense system in maize and is effective in reducing the adverse effects of the accumulation of ROS caused by waterlogging on plant physiological traits (Hu et al., 2020).

Waxy corn, a variety of maize expressing the only amylopectin, has been extensively planted in China and many other countries (Ketthaisong et al., 2014). However, maize (*Zea mays* L.) is considered sensitive to variations in its environment, especially during the seedling stage (Xie et al., 2017; Wang et al., 2018; Babu et al., 2020). In particular, maize is highly sensitive to waterlogging stress, which in the early growth stage, significantly inhibits its growth and development. Waterlogging during the seedling stage causes the greatest delay to growth (Ren et al., 2014), causing the leaves to be withered and yellow, decreasing the maximum green leaf area, reducing photosynthetic efficiency, and exacerbating oxidative damage (Tang et al., 2010; Wu et al., 2018; Yao, 2021). In particular, grain yield decreases most significantly when plants are waterlogged during the seedling stage compared with other stages (Ren et al., 2018b). In addition to heavy rainfall caused by extreme weather, waterlogging due to the lack of drainage in low-lying areas is also a leading factor (Visser et al., 2003; Barik et al., 2019). Therefore, improving tolerance to waterlogging is critical for the production of waxy corn that experiences waterlogging stress. However, to the best of our knowledge, no study has investigated the role of 6-BA-induced tolerance to waterlogging stress in waxy corn. Based on previous studies, which have explored the role of 6-BA in other plants undergoing different forms of stress (Ji et al., 2019; Chen et al., 2020; Hu et al., 2020; Li et al., 2020; Wang X. Y. et al., 2020), we hypothesized that 6-BA scavenges ROS by the promotion of the AsA-GSH cycle system and alleviates the adverse effects of oxidative damage from waterlogging stress in waxy corn seedlings, thereby enhancing tolerance to waterlogging in waxy corn seedlings. Hence, the effect of leaf-sprayed 6-BA in two genotypes of waxy corn seedlings when waterlogged was investigated by comparing morphology, ROS metabolism, cell stability, and the AsA-GSH cycle to verify whether 6-BA prevents oxidative damage from waterlogging stress in waxy corn seedlings. Such an approach may represent a cost-effective and environmentally friendly method to solve the problems of waterlogging stress in waxy corn production.

## Materials and Methods

### Plant Materials and Experimental Design

Pot experiments were conducted at the Research and Education Center of Agronomy, Shenyang Agricultural University (Shenyang, China) in 2020 using two waxy corn inbred lines, namely, SY-MY13 (waterlogging-resistant) and SY-XT1 (waterlogging-sensitive), provided by the Specialty Corn Institute, Shenyang Agricultural University. Pots that were 12 cm in height and 10 cm in diameter were filled with 1 kg of conventional tilled brown soil (from a depth of 0–to 20 cm) with a composition of 32.45 g.kg^−1^ of organic matter, 121.86 of mg.kg^−1^ of alkali-hydrolyzable nitrogen, 9.47 mg.kg^−1^ of available phosphorus, and 114.31 mg.kg^−1^ of available potassium. The experiment was conducted under a rain shelter covered with polyethylene film to exclude any natural precipitation in the experiment. Seeds were sown into each pot on May 12. Three healthy seedlings were retained after germination, with normal watering until the fourth leaf stage. The experiments were conducted using an entirely randomized design. The 6-BA (analytically pure) used in this study was obtained from Ryon Biological Technology Co., Ltd. (Shanghai, China) and dissolved in distilled water containing 0.01% of Tween-20 to a final concentration of 0.5 mM. The concentrations of 6-BA were selected from the results of a previously reported study (Chen et al., 2013). The 6-BA was evenly sprayed on the leaves of waxy corn seedlings. There were four treatment groups for each inbred line, namely, (1) CK: normal watering conditions; (2) CK + 6-BA: 0.5 mM of 6-BA plus normal watering conditions; (3) W: waterlogged; (4) W + 6-BA: 0.5 mM of 6-BA plus waterlogging. Treatments started on June 6. Pots from the same group were placed into plastic boxes (61 × 42 × 12 cm). Prior to starting the waterlogging stress treatments, 0.5 mM of 6-BA was applied as a foliar spray on all the leaves of each treatment group until they were completely moistened. After 24 h, water was added to the plastic boxes to a depth of 3 cm above the soil surface in the waterlogged groups. There were 40 pots in each treatment group. After 7 days, the whole plants and three fully expanded leaves from the bottom of the plants were collected for analysis and measurement. All measurements were performed in triplicates and the mean values were recorded.

### Determination of Growth Parameters

Growth parameters were measured in accordance with an earlier study (Wang et al., 2021). Shoot height and leaf dimensions were measured using a ruler. Leaf area was calculated in accordance with the following formula:

$$\begin{array}{l} \text{Leaf area (c}{\text{m}}^{2}\text{)}=\text{L}\times \text{W}\times 0.75 \end{array}$$

where L represented leaf length, W was leaf width, and 0.75 was a factor used for maize seedlings that consider leaf shape (Hussain et al., 2019).

Each whole plant was subdivided into shoots and roots. Roots were cleaned with tap water to remove adherent soil and then dried with absorbent paper. The fresh weight (FW) of the shoots and roots were individually measured using an analytical balance (*UQINTIX65-1CN*, Sartorius, Göttingen, Germany) and were then loaded into sample bags after 2 h of drying in an oven at 105°C. After that, the shoots and roots were dried to a constant weight at 80°C. The dry weight (DW) of each shoot and root was then measured using the analytical balance.

Roots were placed in an acrylic tray (400 × 300 mm) in a 1 cm depth of water. The length, volume, diameter, and surface area of the roots were measured through scanning using a flatbed scanner (*1680*, Epson, Nagano, Japan) and analysis with the WinRHIZO root analysis software (*Pro 2007*, Regent Instruments, Québec, Canada).

### Quantification of Lipid Peroxidation and Relative Electrical Conductivity

Malondialdehyde content was measured in accordance with a method described by Ohkawa et al. (1979). A total of 0.5 g of fresh leaves were ground and homogenized in 5 ml of 5% trichloroacetic acid (TCA) then centrifuged at 12,000 × *g* for 10 min. The supernatant (2 ml) was retained and mixed with TCA (5%) containing thiobarbituric acid (TBA, 0.5%), placed in a water bath for 20 min at 100°C, then rapidly cooled and centrifuged at 12,000 *g* for 10 min. The absorbances of the supernatant at 450, 532, and 600 nm were recorded using a microplate reader (*1510*, Thermo Fisher, USA). Relative electrical conductivity (REC%) was determined using a conductivity meter (*DDSJ-380F*, Rex, Shanghai, China) and measured in accordance with a method described by Li A. X. et al. (2015). Briefly, 0.1 g of fresh leaves were immersed in deionized water, and the electrical conductivity of the solution was measured after 3 h, termed EC1. The solution was placed in a water bath for 30 min at 100°C, then cooled to room temperature, shaken, and its electrical conductivity measured, termed EC2. REC% was calculated using the following formula:

$$\begin{array}{l} \text{REC}\%=\text{EC}1/\text{EC}2\times 100\% \end{array}$$

### Histochemical Staining and Determination of ${\text{O}}_{2}^{\text{–}}$ and H_2_O_2_

Histochemical staining for ${\text{O}}_{2}^{-}$ and H_2_O_2_ was conducted in accordance with a method described by Xia et al. (2009), with modifications. The three lowest fully expanded leaves were washed with distilled water, blotted dry, then placed into a test tube containing 50 ml of 0.5 mg/ml nitrotetrazolium blue chloride (NBT) and a reaction solution (potassium phosphate buffer, pH 7.8), respectively, prior to incubation at 25°C in the dark for 2 h. Leaves were also placed in 50 ml of 1 mg/ml diaminobenzidine (DAB) reaction solution (Tris-HCl buffer, pH 3.8), which was incubated at 25°C in the dark for 24 h. The solutions in the individual tubes were replaced with a bleaching solution (90% ethanol) and placed in a water bath at 90°C for 15 min until the leaves were fully bleached; after which, they were photographed.

To measure ${\text{O}}_{2}^{-}$ and H_2_O_2_, a method described by Xu et al. (2012) was followed. A total of 0.5 g of fresh leaves were ground in liquid nitrogen, suspended in 50 mM of phosphate-buffered saline (PBS, pH 7.8), and then centrifuged at 12,000 × *g* for 15 min. The concentration of ${\text{O}}_{2}^{-}$ in the supernatant was measured using a microplate reader (*1510*, Thermo Fisher Scientific, Waltham, MA, USA) at a wavelength of 580 nm. For measurements of H_2_O_2_, 0.5 g of fresh leaves were ground in liquid nitrogen, suspended in 100 mM of PBS (pH 7.8) containing 1% (w/v) polyvinyl pyrrolidone (PVP), and then centrifuged at 12,000 × *g* for 20 min. The concentration of H_2_O_2_ in the supernatant was measured using a microplate reader at a wavelength of 350 nm.

### Measurements of ASA-GSH Cycle Enzyme Activity

A 0.5-g quantity of fresh leaves was ground in liquid nitrogen, then homogenized in 10 ml of an extraction buffer (pH 7.8) containing 25 mM of 4-(2-hydroxyethyl)-1-piperazineethanesulfonic acid (HEPES), 2% PVP, 2 mM of ascorbic acid, and 0.2 mM of ethylenediaminetetraacetic acid (EDTA). The homogenized solution was centrifuged at 12,000 × *g* for 20 min at 4°C. The activity of the AsA-GSH cycle enzymes in the supernatant was measured. The activities of APX and DHAR were calculated in accordance with a method published by Nakano and Asada (1980), GR activity was calculated in accordance with GarcíaLimones et al. (2002), and MDHAR activity was calculated in accordance with (Aravind and Prasad, 2005).

### Measurements of Concentration of AsA-GSH Cycle Metabolites

A 0.5-g quantity of fresh leaves was ground in liquid nitrogen and then homogenized in 10 ml 10% (w/v) trichloroacetic acid. The suspension was centrifuged at 15,000 × *g* for 15 min at 4°C. The concentrations of AsA, DHA, GSH, and GSSG were measured in the supernatant. In particular, AsA and DHA concentrations were quantified using a dithiothreitol assay (Hodges et al., 1996) against calibration curves prepared from AsA and DHA standards. Concentrations of GSH and GSSG were measured using a 2-vinylpyridine assay (Griffith, 1980) against calibration curves constructed using GSH and GSSG standards.

### Statistical Analysis

Data were analyzed using a one-way ANOVA, after which, a least significant difference (LSD) test was conducted. Differences were considered significant for *P* < 0.05. Analysis was performed with the *SPSS v19.0 software* (*SPSS Inc*., Chicago, IL, USA). Data were plotted using the Origin 2017 software (*OriginLab*, Massachusetts, USA).

## Results

### Plant Morphology

**Figure 1:**
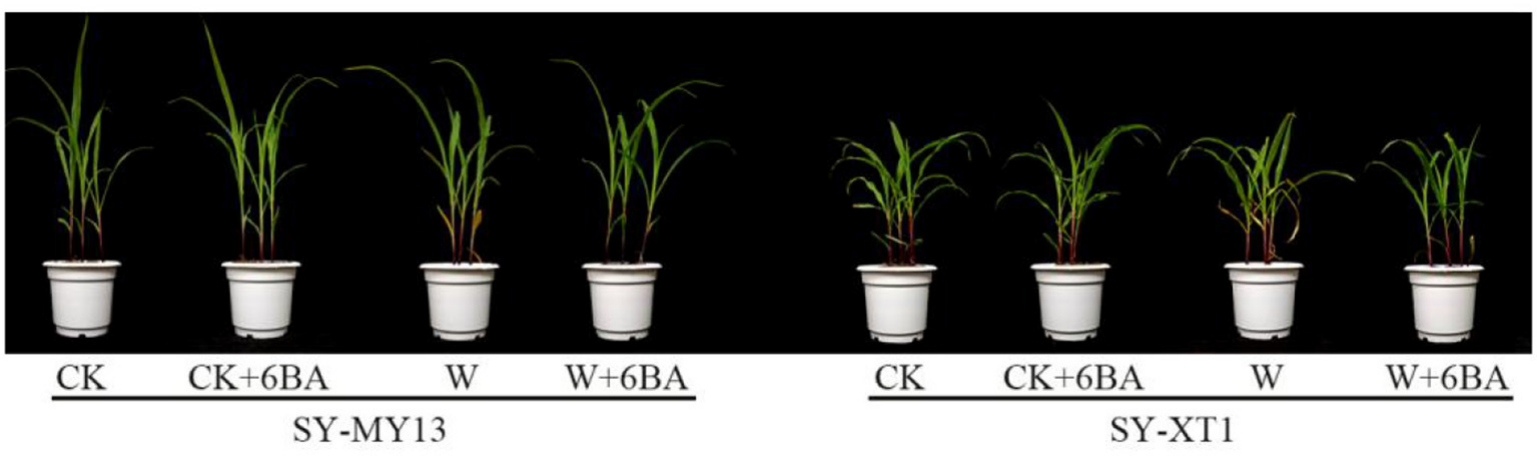
Effects of 6-BA on the phenotype of two inbred lines with different treatments. CK, normal watering conditions only; CK + 6-BA, 0.5 mM of 6-BA plus normal watering conditions; W, waterlogged; W + 6-BA, addition of 0.5 mM of 6-BA plus waterlogging stress conditions.

When grown in normal conditions, plants in the CK and 6-BA groups displayed no apparent differences, as illustrated in Figure 1. Waterlogging stress injuries in the waxy corn seedlings were characterized by chlorosis, wilting, and necrosis. However, the extent of the symptoms was considerably reduced in the SY-MY13 plants due to their excellent waterlogging resistance properties. Plants treated with 6-BA when stressed with waterlogging, on the other hand, did not exhibit visible damage in either inbred lines.

### Plant Growth and Development

The growth parameters directly reflected the adaptability of the plants to the growing environment (Chen D. Q. et al., 2016; Kosar et al., 2021), as presented in Tables 1, 2. Treatment with 6-BA significantly increased the shoot height of SY-MY13 plants, although there were no significant differences for other growth parameters between the two inbred lines in well-drained conditions. Waterlogging significantly reduced the fresh and dry shoot weight of the SY-XT1 plants but had no significant effect on the SY-MY13 strain. Leaf area and fresh and dry root weight were reduced by 17.9, 31.8, and 48.1% in the SY-MY13 strain and 31.5, 49.9, and 59.4% in the SY-XT1 plants compared with those of the CK group, respectively. Similarly, root length and surface area were reduced by 32 and 10.4% in SY-MY13 plants and 22.8 and 12% in SY-XT1 plants, respectively. In addition, the root diameter of the two inbred lines increased by 21.7 and 13.4%, respectively. Treatment with 6-BA also significantly enhanced the growth and development of both waxy corn inbred lines exposed to waterlogging stress. The dry weight of shoots, plant height, leaf area, and root diameter increased by 15.7, 9.8, 15, and 10.4% in SY-MY13 and 47.4, 9.9, 26.3, and 14.9% in SY-XT1 plants treated with 6-BA when waterlogged, respectively. The same treatment also increased the fresh and dry weight of roots by 31.9 and 59.1%, respectively, in SY-XT1 compared with waterlogging stress.

**Table 1:** Effects of 6-benzyladenine (6-BA) on the biomass of two waxy corn inbred lines experiencing waterlogging stress.

**Genotype**	**Treatment**	**Shoot fresh weight (g)**	**Shoot dry weight (g)**	**Root fresh weight (g)**	**Root dry weight (g)**
SY-MY13	CK	3.78 ± 0.208cd	0.522 ± 0.019c	2.64 ± 0.257a	0.239 ± 0.012a
	CK+6BA	3.93 ± 0.100bc	0.535 ± 0.008c	2.33 ± 0.290ab	0.242 ± 0.021a
	W	3.61 ± 0.253d	0.538 ± 0.006c	1.80 ± 0.164c	0.124 ± 0.023b
	W+6BA	3.80 ± 0.227cd	0.638 ± 0.021a	1.16 ± 0.148d	0.139 ± 0.018b
SY-XT1	CK	4.17 ± 0.088ab	0.547 ± 0.013bc	2.24 ± 0.240b	0.229 ± 0.024a
	CK+6BA	4.27 ± 0.228a	0.544 ± 0.036bc	2.43 ± 0.050ab	0.237 ± 0.014a
	W	2.73 ± 0.100e	0.304 ± 0.01d	1.13 ± 0.062d	0.093 ± 0.007c
	W+6BA	3.75 ± 0.114cd	0.578 ± 0.029b	1.49 ± 0.107c	0.148 ± 0.014b

*CK, normal watering conditions only; CK + 6-BA, 0.5 mM of 6-BA plus normal watering conditions; W, waterlogged; W + 6-BA, addition of 0.5 mM of 6-BA plus waterlogging stress conditions. Data represent means ± SD of three replicates. For each variable, means with different lowercase letters represent significant differences (P < 0.05)*.

**Table 2:** Effects of 6-BA on the growth of two waxy corn inbred lines experiencing waterlogging stress.

**Genotype**	**Treatment**	**Shoot height (cm)**	**Leaf area (cm^**2**^)**	**Root length (cm)**	**Root surface area (cm^**2**^)**
SY-MY13	CK	45.0 ± 2.646b	153.2 ± 18.292bc	2,114.7 ± 259.427a	494.7 ± 50.615a
	CK+6BA	49.2 ± 0.289a	160.3 ± 11.884bc	2,093.2 ± 128.438a	496.5 ± 7.904a
	W	43.3 ± 1.155b	125.6 ± 4.346d	1,438.0 ± 13.169bc	443.1 ± 7.057bcd
	W+6BA	48.0 ± 1.732a	147.8 ± 9.464c	1,217.1 ± 97.011cd	417.8 ± 14.544cd
SY-XT1	CK	37.8 ± 0.289cd	181.6 ± 6.255a	1,695.6 ± 193.975b	467.9 ± 21.746ab
	CK+6BA	38.8 ± 0.764cd	184.0 ± 4.611a	1,613.5 ± 222.672b	457.2 ± 34.470abc
	W	36.3 ± 2.081d	124.5 ± 3.243d	1,309.5 ± 38.747cd	411.8 ± 3.076d
	W+6BA	40.3 ± 1.528c	168.9 ± 3.175ab	1,096.2 ± 164.101d	401.0 ± 24.899d

*CK, normal watering condition only; CK + 6-BA, 0.5 mM of 6-BA plus normal watering conditions; W, waterlogged; W + 6-BA, addition of 0.5 mM of 6-BA plus waterlogging stress conditions. Data represent means ± SD of three replicates. For each variable, means with different lowercase letters represent significant differences (P < 0.05)*.

### Oxidative Stress and Histochemical Staining

**Figure 2:**
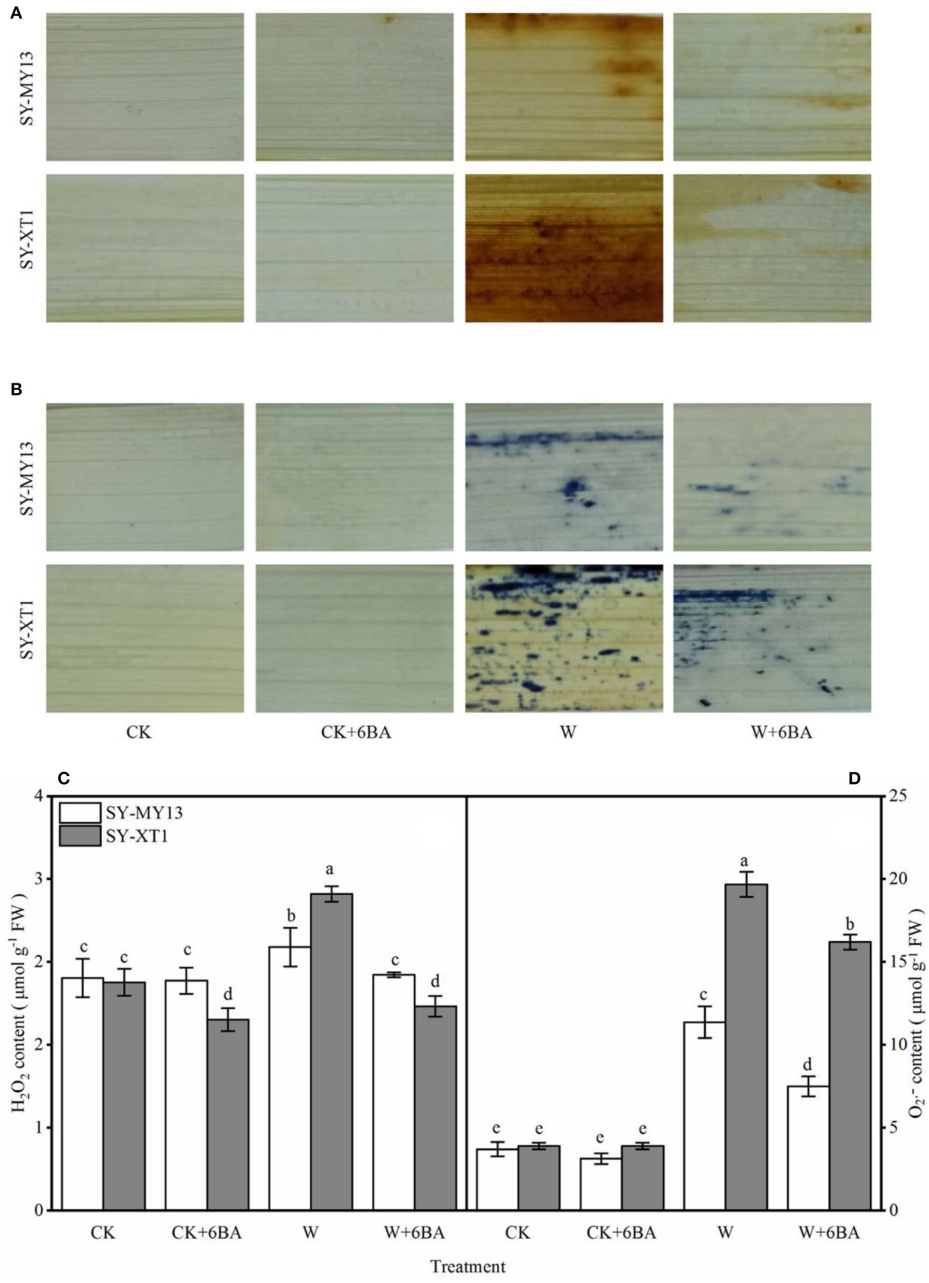
Effects of 6-BA on histochemical staining of H_2_O_2_ **(A)** and ${\text{O}}_{2}^{-}$ **(B)**, Concentration of H_2_O_2_ **(C)** and ${\text{O}}_{2}^{-}$ **(D)** in two waxy corn inbred lines in waterlogged conditions. CK, normal watering conditions only; CK + 6-BA, 0.5 mM of 6-BA plus normal watering conditions; W, waterlogged; W + 6-BA, Addition of 0.5 mM of 6-BA plus waterlogging stress conditions. Data represent means ± SD of three replicates. For each variable, means with different lowercase letters were significantly different (*P* < 0.05).

Histochemical staining techniques have previously been used to detect H_2_O_2_ and ${\text{O}}_{2}^{-}$ in plants (Xu et al., 2012). Compared with CK, the CK + 6-BA group displayed a significantly lower H_2_O_2_ content in SY-XT1, but displayed no significant difference in SY-MY13 plants (Figure 2C). In addition, there was no significant difference in the changes in ${\text{O}}_{2}^{-}$ content in the CK and CK + 6-BA groups between the two inbred lines (Figure 2D). The histochemical staining of the leaves also revealed that there was less H_2_O_2_ and ${\text{O}}_{2}^{-}$ accumulation when plants were waterlogged following pretreatment with 6-BA compared with waterlogged plants that were untreated (Figures 2A,B). As shown in Figures 2C,D, the leaves of the two inbred lines displayed higher levels of H_2_O_2_ and ${\text{O}}_{2}^{-}$ accumulation when waterlogged compared with those that were well-drained, with H_2_O_2_ and ${\text{O}}_{2}^{-}$ levels in SY-MY13 significantly increasing by 13.6 and 208.1% and in SY-XT1 by 40.9 and 405.1%, respectively, compared with the CK groups. Pretreatment with 6-BA resulted in a significant decrease in both H_2_O_2_ and ${\text{O}}_{2}^{-}$ levels in the SY-MY13 plants, reducing by 10.2 and 34% and in SY-XT1 by 35.6 and 17.6%, respectively, compared with those that were waterlogged (Figures 2A–D).

### Oxidative Damage to Cellular Membranes

**Figure 3:**
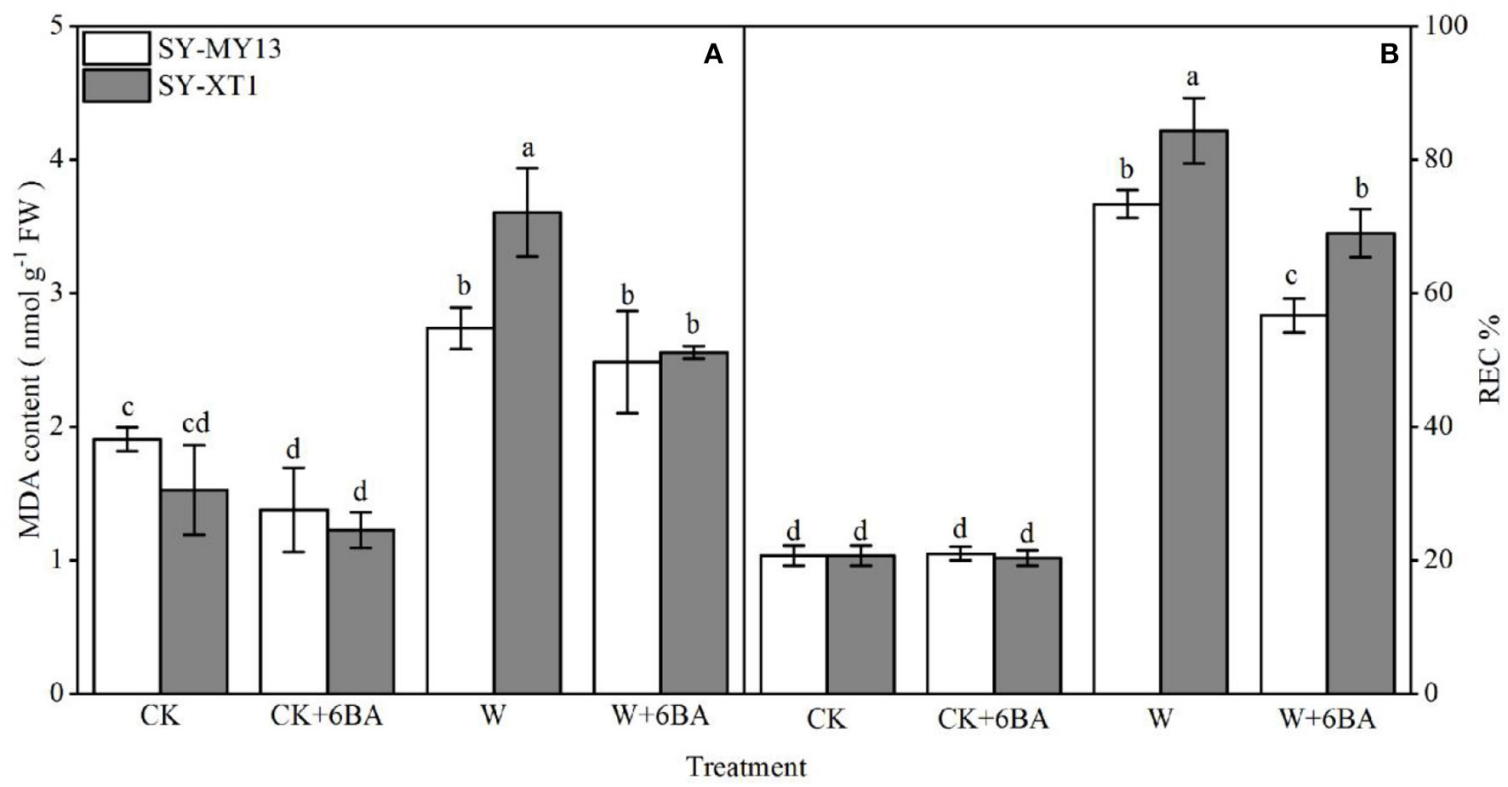
Effects of 6-BA on malondialdehyde (MDA) concentration **(A)** and relative electric conductivity (REC%) **(B)** in two waxy corn inbred lines experiencing waterlogging. CK, normal watering conditions only; CK + 6-BA, 0.5 mM of 6-BA plus normal watering conditions; W, waterlogged; W + 6-BA, addition of 0.5 mM of 6-BA plus waterlogging stress conditions. Data represent means ± SD of three replicates. For each variable, means with different lowercase letters were significantly different (*P* < 0.05).

Both MDA concentration and REC% reflect the extent of damage to cell membranes (Farooq et al., 2020). As displayed in Figure 3, pretreatment with 6-BA in plants that were watered normally exhibited a significantly reduced MDA concentration in SY-MY13 plants, but showed no effect on SY-XT1. It also had no significant effect on REC% in either of the two inbred lines. The MDA content and REC% increased in both inbred lines when the plants were waterlogged compared with the CK groups, although the increase was greater in SY-XT1 plants (140 and 307.2%) compared with SY-MY13 (42.1 and 254.1%). Furthermore, pretreatment with 6-BA resulted in a significant decrease in MDA levels and REC% compared with those that were waterlogged. The MDA concentration and REC% of SY-MY13 only increased by 9.5 and 22.6%, with the same observed in SY-XT1 by 27.8 and 17.9%, respectively.

### Enzymatic and Non-enzymatic Metabolites of the AsA-GSH Cycle

**Figure 4:**
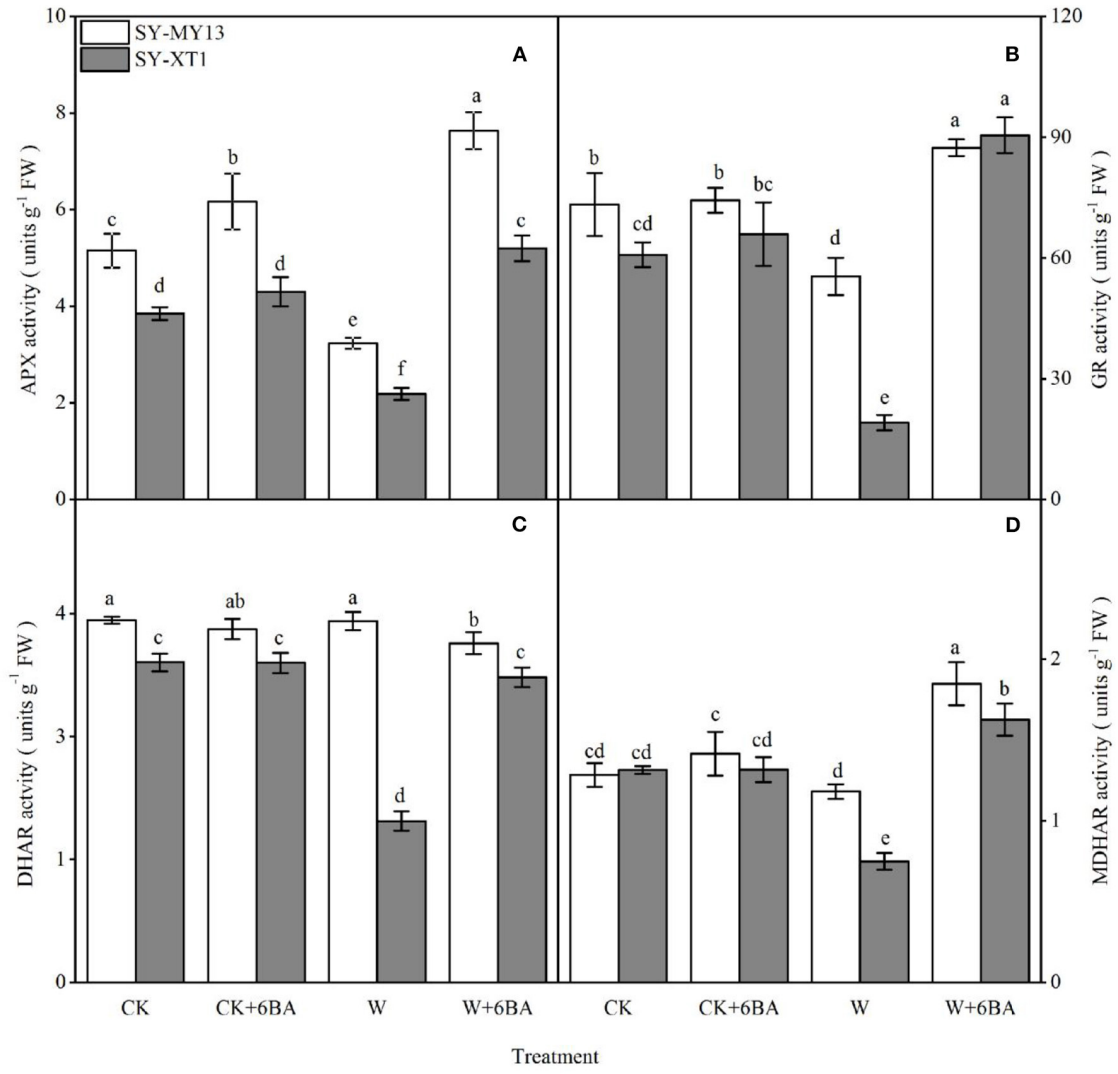
Effects of 6-BA on ascorbate peroxidase (APX) **(A)**, glutathione reductase (GR) **(B)**, dehydroascorbate reductase (DHAR) **(C)**, and monodehydroascorbate reductase (MDHAR) **(D)** activity in two waxy corn inbred lines experiencing waterlogging. CK, normal watering conditions only; CK + 6-BA, 0.5 mM of 6-BA plus normal watering conditions; W, waterlogged; W + 6-BA, addition of 0.5 mM of 6-BA plus waterlogging stress conditions. Data represent means ± SD of three replicates. For each variable, means with different lowercase letters were significantly different (*P* < 0.05).

By using the enzymatic defense system in the AsA-GSH cycle, plants can scavenge ROS generated by stress conditions (Niu et al., 2018; Abd-Allah et al., 2019). As identified in the two inbred lines, spraying 6-BA increased APX activity compared with well-drained plants, although the difference was only significant for SY-MY13. In addition, spraying 6-BA slightly elevated GR and MDHAR activity in the two inbred lines compared with well-drained plants, although the differences were not significant (Figure 4). Thus, waterlogging clearly affects enzymes in the AsA-GSH cycle, negatively. Furthermore, the activity of APX and GR decreased significantly compared with the CK group when waterlogged in SY-MY13 by 37.3 and 24.4% and SY-XT1 plants by 43.4 and 68.6%, respectively (Figures 4A,B). The activity of DHAR and MDHAR in SY-XT1 plants declined significantly, by 50 and 43%, while there were no significant differences in DHAR and MDHAR activity in SY-MY13 plants (Figures 4C,D). Pretreatment with 6-BA for the two waxy corn inbred lines exposed to waterlogging stress conditions also resulted in an increasing trend in the activity of AsA-GSH cycle enzymes relative to that of non-sprayed plants. The activity of APX, GR, and MDHAR increased significantly in SY-MY13 and SY-XT1 plants by 137.5, 57.8, and 60% and 136.4, 373.8, and 116.9%, respectively (Figures 4A,B,D). The activity of DHAR in SY-XT1 plants also increased significantly by 94.4% (Figure 4C).

**Figure 5:**
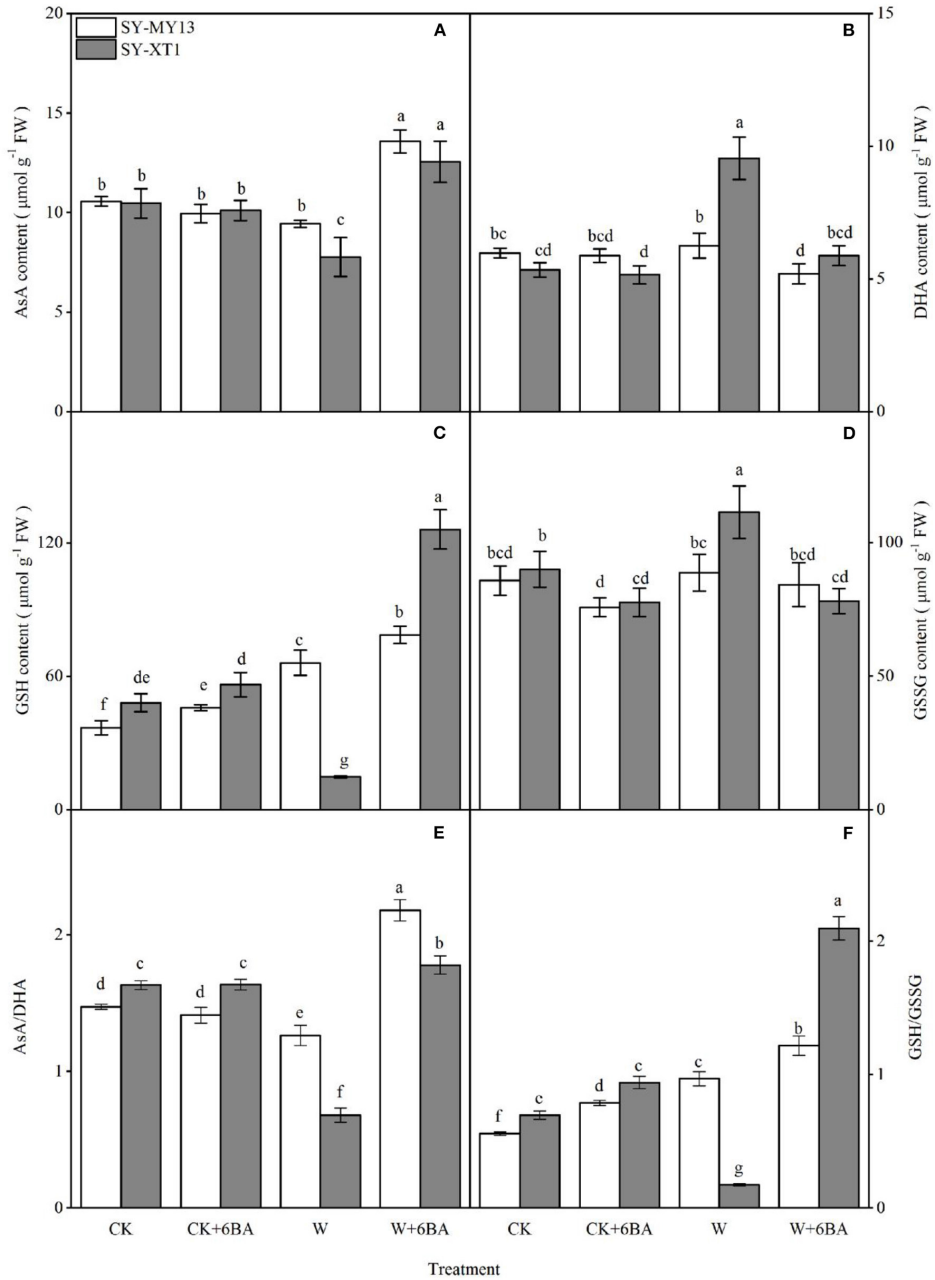
Effects of 6-BA on ascorbic acid (ASA) **(A)**, dehydroascorbic acid (DHA) **(B)**, reduced glutathione (GSH) **(C)**, and oxidized glutathione (GSSG) concentrations **(D)** and the ratios AsA/DHA **(E)** and GSH/GSSG **(F)** in two waxy corn inbred lines in waterlogged conditions. CK, normal watering conditions only; CK + 6-BA, 0.5 mM of 6-BA plus normal watering conditions; W, waterlogged; W + 6-BA, addition of 0.5 mM of 6-BA plus waterlogging stress conditions. Data represent means ± SD of three replicates. For each variable, means with different lowercase letters were significantly different (*P* < 0.05).

The metabolites in the AsA-GSH cycle can both directly scavenge ROS and act as enzymatic substrates that reduce ROS (Raja et al., 2020). The concentrations of AsA, GSH, and their oxidized forms for the different treatments are displayed in Figures 5A–D. Spraying 6-BA in well-drained plants increased the GSH content of the two inbred lines and decreased the GSSG content of SY-XT1. Higher ratios of GSH/GSSG were accompanied by changes in AsA and GSH pools in SY-MY13 and SY-XT1 plants. Waterlogging increased GSH content in SY-MY13 plants by 79.6% but affected SY-XT1 to a greater extent, with AsA and GSH levels decreasing by 25.7 and 69.2% and those of DHA and GSSG increasing by 79.2 and 23.8%. Thus, there was a significant decline in the AsA/DHA ratio, by 14.7 and 58.7%. In contrast, the GSH/GSSH ratio in SY-MY13 plants increased by 74.4%, while it decreased by 75.5% in SY-XT1 plants. Furthermore, 6-BA pretreatment enhanced AsA and GSH content and reduced DHA and GSSG levels when plants were waterlogged. The levels of AsA and GSH in SY-MY13 plants increased by 44.7 and 18.9% and in SY-XT1 by 61.5 and 751.4%, respectively. The levels of DHA and GSSG in the SY-MY13 strain decreased by 16.1 and 5% and in SY-XT1 by 37.9 and 42.8%, respectively. Treatment with 6-BA significantly increased AsA/DHA and GSH/GSSH ratios compared with waterlogging stress treatment, with these ratios in the 6-BA-sprayed groups increasing significantly when compared with the non-sprayed groups when waterlogged in SY-MY13 plants, by 72.6 and 25.6% and in SY-XT1 by 162.6 and 111.4%, respectively.

## Discussion

Waterlogging stress adversely affects the growth of many terrestrial crops, causing chlorosis and necrosis in their leaves, inhibiting shoot and root growth, and decreasing dry matter accumulation (Komatsu et al., 2013; Wang et al., 2017; Kaya et al., 2019; Men et al., 2020). The inhibitory effects of waterlogging stress on plant growth and development are likely due to hypoxic conditions in the root zone that hamper root respiration, resulting in limited nutrient uptake and transport (Tan et al., 2010; Qi et al., 2016; Wang et al., 2019). In the present study, waterlogging stress injuries in waxy corn seedlings were characterized by chlorosis, wilting, and necrosis (Figure 1), which cause the repression of seedling growth (Tables 1, 2) as confirmed by Zhu et al. (2016). In addition, waterlogging also significantly reduced the fresh and dry weight of shoots in SY-XT1 plants, while there was no significant effect on SY-MY13, which may be due to the waterlogging-sensitive line more readily losing biomass when exposed to waterlogged conditions (Striker, 2012). Furthermore, cytokinin content decreased when plants were placed in stressful conditions, which represents a limiting factor for the growth of plants (Shams and Yildirim, 2021). Cytokinins allow the optimal growth and development of plants by promoting cell division and tissue growth and delaying leaf senescence (Davies, 2010; Kim et al., 2018). Furthermore, 6-BA is in a class of synthetic cytokinin PGRs that can significantly increase levels of endogenous cytokinins in crop plants (Hu et al., 2020; Prerostova et al., 2020). In the present study, after spraying with 6-BA, the two waxy corn inbred lines exhibited reduced injuries in terms of growth and had greater leaf area, shoot height, and dry weight compared with the non-sprayed plants when waterlogged. This proves the hypothesis that 6-BA acts positively to regulate the response to waterlogging stress in waxy corn seedlings. Plant biomass has also been shown to increase, for example, by the application of PGRs capable of producing growth hormones (Cohen et al., 2009). Therefore, the exogenous application of 6-BA can mitigate the adverse effects of waterlogging stress on plant growth.

Waterlogging results in low O_2_ levels in plant tissues that can, in turn, lead to excess ROS production *via* the disruption of the balance of ROS generation and detoxification (Posso et al., 2018; Anee et al., 2019; Park and Ju, 2019). The first ROS to be generated is usually ${\text{O}}_{2}^{-}$, with the radicals forming H_2_O_2_ spontaneously by dismutation (Mori and Schroeder, 2004). Excess H_2_O_2_ production when plants are waterlogged affects multiple physiological processes because H_2_O_2_ is a strong uncharged oxidant molecule (Castro-Duque et al., 2020; Cen et al., 2020). Waterlogging stress inflicts significant damage on waxy corn seedlings, as suggested by the greater levels of ${\text{O}}_{2}^{-}$ and H_2_O_2_ in the leaves of both the SY-MY13 and SY-XT1 inbred lines used in the current study (Figures 2C,D), which also demonstrated the histochemical staining of ${\text{O}}_{2}^{-}$ and H_2_O_2_ at the tissue level in the leaves of waxy corn (Figures 2A,B). The ROS injury of the bio membranes also causes lipid peroxidation and disrupts membrane homeostasis (Shu et al., 2012; Song et al., 2021). In the study, the waterlogging stress induction of ROS causing membrane damage was also reflected by the increased MDA content and REC% (Figure 3). The application of exogenous 6-BA reduced damage in the seedlings when waterlogged by significantly reducing the waterlogging-induced production of H_2_O_2_ (Figure 2C), ${\text{O}}_{2}^{-}$ (Figure 2D), MDA (Figure 3A), and REC% (Figure 3B) compared with those of waterlogging alone. Moreover, histochemical staining indicated that the levels of ${\text{O}}_{2}^{-}$ and H_2_O_2_ in tissues were lower in the leaves (Figures 2A,B). Furthermore, endogenous cytokinin levels when plants experience environmental stress can be enhanced by the application of synthetic cytokinins, which can offset stress-induced premature senescence of plants, in addition to reducing damage due to ROS and lipid peroxidation; thus, effectively improving the adaptability of plants experiencing stress (Gujjar and Supaibulwatana, 2019). Our studies revealed that pretreatment with 6-BA not only decreased MDA content but also suppressed ROS accumulation and electrolyte leakage in waxy corn seedlings when exposed to waterlogging stress. These findings demonstrated that 6-BA has excellent potential for use in applications that maintain the integrity of cellular membranes through the prevention of lipid peroxidation against waterlogging-induced oxidative damage, which is a possible principal mechanism by which waterlogging stress is alleviated in maize plants. Interactions between cytokinin signaling and ROS production and scavenging systems have also been demonstrated in arabidopsis (Nishiyama et al., 2012). Feng et al. (2004) also demonstrated that 6-BA can significantly reduce oxidative damage in poplar, the rate of ${\text{O}}_{2}^{-}$ generation, and H_2_O_2_ and MDA levels becoming effectively reduced, indicating that reduced oxidative damage due to 6-BA was associated with the promotion of the scavenging system for ROS. Ren et al. (2018a) reported that exogenous 6-BA increases the tolerance of maize seedlings to waterlogging stress by the protection of physiological processes from oxidative damage and enhancement of the metabolism of ROS. These findings suggest that exogenous 6-BA enhances the tolerance of waxy corn seedlings to waterlogging by the attenuation of ROS-induced oxidative damage.

Waterlogging stress-induced hypoxic injury generally results in oxidative stress by inducing the production of ROS. The antioxidant system in plants plays a critical role in ROS scavenging and is positively correlated with waterlogging tolerance (Da-Silva and Do-Amarante, 2020; Mira et al., 2021). Ascorbate peroxidase is a pivotal enzyme in the AsA-GSH cycle; since, it utilizes AsA as a substrate to reduce H_2_O_2_ to H_2_O (Gordon et al., 2013; Ghosh and Biswas, 2017; Mir et al., 2018). Glutathione reductase is also involved in the defense against oxidative stress, in addition to the regeneration of GSH (Kaur et al., 2018; Guo et al., 2019). Furthermore, both DHAR and MDHAR are key to maintaining AsA concentration (Zhang et al., 2015; Juszczak et al., 2016; Raja et al., 2020). In the present study, the AsA-GSH cycle was activated when SY-MY13 plants were exposed to waterlogging stress, enabling normal metabolic activity through exposure to excess ROS. In contrast, the AsA-GSH cycle of SY-XT1 plants operated at a relatively low level, and so were more vulnerable to ROS damage. Thus, the current data indicate that plants with higher efficiencies in the AsA-GSH cycle can resist waterlogging stress that can be further increased by 6-BA supplementation in waxy corn plants. In the present study, increased APX, GR, DHAR, and MDHAR activity were observed in waxy corn seedlings supplemented with 6-BA exposed to waterlogging stress (Figure 4). Improvements in the tolerance of waxy corn to waterlogging are associated with a reduction in H_2_O_2_ content and attributable to the enhanced activity of APX. In addition, increased MDHAR activity suggests that it can act synergistically with APX to increase the efficiency of the AsA-GSH cycle and maintain the redox status and antioxidant activity of the AsA pool. These results are further supported by the observations of Jiang et al. (2019) and Prerostova et al. (2020), who observed that high levels of cytokinins positively affect environmental stress tolerance in arabidopsis due to the cytokinins preventing oxidative stress by the activation of APX, which removes excess ROS. In a study by Porcher et al. (2021), cytokinins were also shown to act, in part, through the induction of the AsA-GSH scavenging pathways, which decrease internal H_2_O_2_ concentrations in buds that were observed during bud bursting events, thereby regulating ROS homeostasis in the buds of roses. A study by Chen and Yang (2013) also found that APX and GR activity in cucumber fruits treated with 6-BA increased after suffering chill injuries by reducing increased membrane permeability and lipid peroxidation, delaying increases in ${\text{O}}_{2}^{-}$ and H_2_O_2_ levels, and maintaining higher levels of total antioxidant capacity. The application of 6-BA led to the increased activity of the AsA-GSH cycle enzymes when experiencing environmental stress-induced oxidative damage, as has been previously observed in tomatoes and eggplants (Chen J. L. et al., 2016; Singh et al., 2018). The results of the present study reveal that exogenous 6-BA is able to coordinate relative internal balance and the high activity of APX, GR, DHAR, and MDHAR, thus allowing adaptation to stress and enabling the balance between the production and scavenging of ROS through the modulation of the levels of the AsA-GSH cycle enzymes. This provides a strong guarantee that AsA and GSH highly efficient AsA-GSH cycle enzymes that are effective in removing ROS caused by waterlogging stress, can be regenerated, thus reducing ROS-induced oxidative damage, subsequently relieving the deleterious effects caused by waterlogging stress, and enhancing the waterlogging tolerance of waxy corn seedlings.

Ascorbic acid and GSH are the most abundant hydrophilic non-enzymatic antioxidants in cells and play a critical role in reducing oxidative stress induced by waterlogging (Pulido et al., 2010; Bai et al., 2013). As a substrate, AsA scavenges H_2_O_2_ and prevents lipid peroxidation (Noctor et al., 2018). In contrast, GSH is a thiol-based antioxidant that prevents lipid peroxidation and contributes to AsA regeneration (Foyer and Halliwell, 1976; Racz et al., 2020). Furthermore, both DHA and GSSG are oxidized forms of AsA and GSH, respectively. The results of the present study indicate that waterlogging decreases AsA and GSH levels but elevates DHA and GSSG in waxy corn seedlings, relative to untreated plants, showing that waterlogging stress clearly leads to a modified oxidation-reduction status in cells through interaction with pools of AsA and GSH (Figure 5). This is due to reductive molecules (AsA and GSH) being involved in the elimination of ROS, resulting in the increased consumption of the AsA and GSH pools. Moreover, decreased DHAR and MDHAR activities under waterlogging stress also resulted in the lack of supplementation to AsA and GSH pools (Figures 4C,D), which leading to the accumulation of a large quantity of oxidized molecules (DHA and GSSG). Li Z. et al. (2015) considered that the GSH/GSSG ratio increased with increasing cytokinin concentration, allowing the maintenance of higher levels of antioxidants, particularly AsA, and thus enhancing the antioxidant capacity of plants. Therefore, the interplay between cytokinins and the AsA-GSH cycle may represent the mechanism by which environmental tolerance is regulated in plants. The AsA-GSH cycle is an efficient antioxidant system for the elimination of excessive ROS production through the maintenance of the AsA/DHA and GSH/GSSG ratios. The data in the present study demonstrate that the AsA/DHA and GSH/GSSH ratios of the waterlogging-sensitive line were both significantly lower than those of CK plants, suggesting that waterlogging stress breaks the balance between the reduced and oxidized forms of AsA and GSH in the AsA-GSH cycle. The concentrations of AsA and GSH and the AsA/DHA and GSH/GSSG ratios increased when 6-BA was applied exogenously to seedlings affected by waterlogging stress (Figures 5A,C,E,F), helping to reduce the ROS responsible for oxidative damage, which is reflected in the reduced levels of MDA and REC% (Figure 3). Recent investigations have demonstrated that GSH levels increase substantially in plants to which cytokinins were added compared with untreated controls (Porcher et al., 2021). Similar results have been demonstrated in studies on exogenous 6-BA applied to *Solanum melongena* after suffering oxidative damage (Wu et al., 2015). The present study illustrated that exogenous 6-BA reinstates the reductive status of AsA and GSH pools, which enhances the activity of the AsA-GSH cycle and allows a reduction in the ROS that cause oxidative damage, and enhances the tolerance of waxy corn seedling to waterlogging stress.

**Figure 6:**
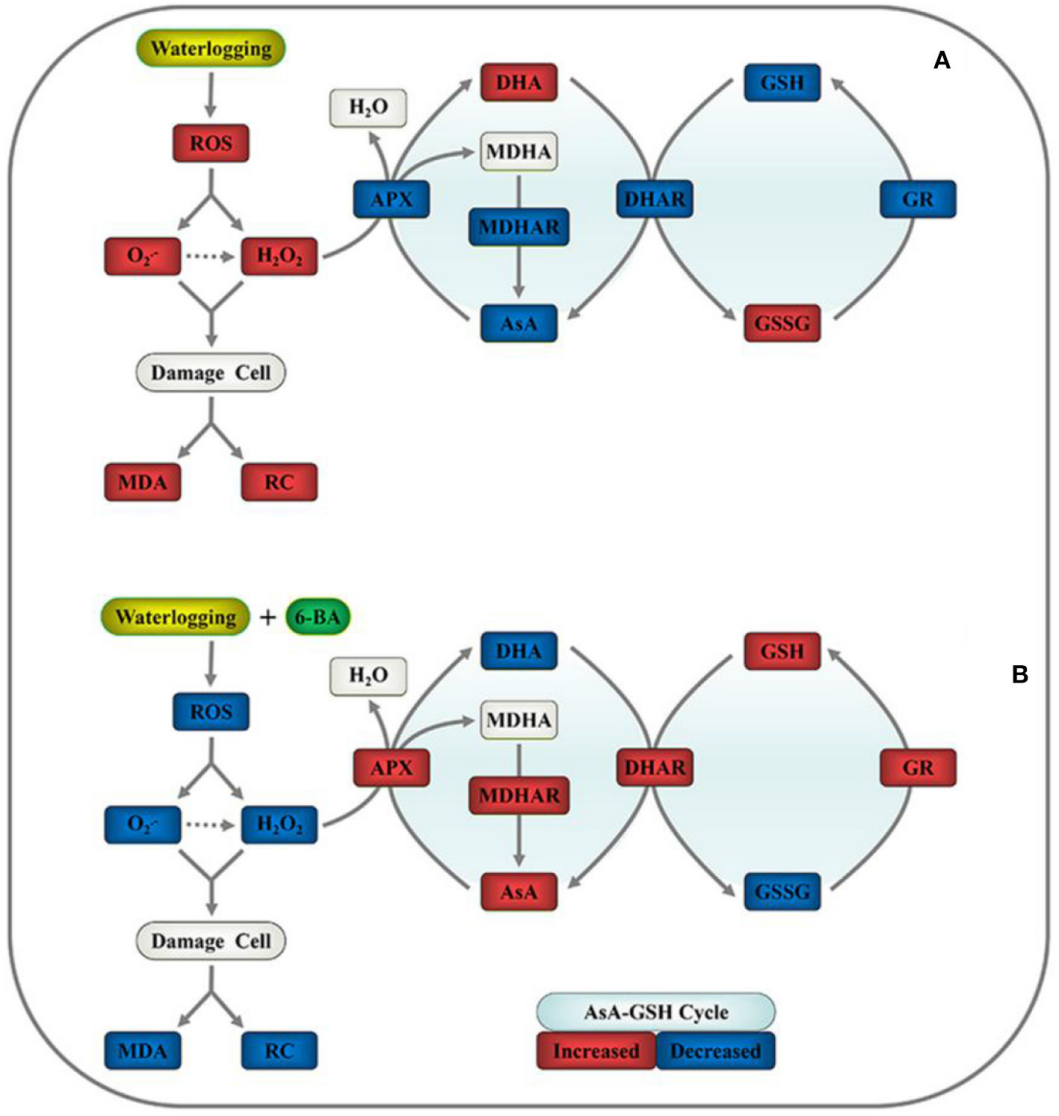
Proposed mechanism for 6-BA-induced waterlogging tolerance in waxy corn seedlings through the mitigation of oxidative stress and sustaining homeostasis of the ascorbate-glutathione (AsA-GSH) cycle. **(A)** Plant growth in waterlogged conditions and **(B)** plant growth after the application of 0.5 mM of 6-BA in waterlogged conditions. Red indicates upregulation, and blue indicates downregulation.

Figure 6 proposes a simple mechanism by which exogenous 6-BA mitigates waterlogging stress in waxy corn seedlings by reducing ROS-induced oxidative stress and sustaining the homeostasis of the AsA-GSH cycle. The application of exogenous 6-BA to waxy corn seedlings when experiencing waterlogging stress significantly decreased the production of ${\text{O}}_{2}^{-}$, H_2_O_2_, MDA, and REC% values relative to those observed in seedlings subjected to waterlogging stress alone. As presented above, the proposed mechanism indicates that the application of 6-BA to waxy corn seedlings when experiencing waterlogging stress promotes AsA-GSH cycle activity, thereby contributing to decreased ROS accumulation in plants, alleviating lipid peroxidation of cell membranes, and maintaining their stability, which also increases waterlogging tolerance in waxy corn seedlings.

## Conclusion

The present data clearly demonstrated that waterlogging stress causes chlorosis and necrosis in waxy corn leaves, subsequently inhibiting growth and leading to ROS accumulation, which induces oxidative stress resulting in membrane lipid peroxidation and the disruption of membrane homeostasis. Exogenous 6-BA ameliorates waterlogging-induced oxidative stress in waxy corn seedlings by stimulating the components of the AsA-GSH cycle and maximally improving the adaptation of waxy corn seedlings to waterlogging stress, thus providing a cost-effective and environmentally friendly method of sustainable crop production, especially in areas vulnerable to waterlogging stress. Therefore, the results reported in this study provided invaluable insights into the critical role of 6-BA in the regulation of the AsA-GSH cycle system and a better understanding of waterlogging tolerance in waxy corn seedlings.

## Data Availability Statement

The original contributions presented in the study are included in the article/supplementary material, further inquiries can be directed to the corresponding author/s.

## Author Contributions

FL and MZ conceived and designed the study, obtained financial support, provided the study material, and helped revise the manuscript. JW performed the experiments, collected data, analyzed data, interpreted data, and maintenance drafted the manuscript. DW helped perform the experiments and participated in the discussion. All authors contributed to the article and approved the submitted version.

## Conflict of Interest

The authors declare that the research was conducted in the absence of any commercial or financial relationships that could be construed as a potential conflict of interest.

## Publisher's Note

All claims expressed in this article are solely those of the authors and do not necessarily represent those of their affiliated organizations, or those of the publisher, the editors and the reviewers. Any product that may be evaluated in this article, or claim that may be made by its manufacturer, is not guaranteed or endorsed by the publisher.

## Abbreviations

AsA-GSH: ascorbate-glutathione
ROS: reactive oxygen species
MDA: malondialdehyde
APX: ascorbate peroxidase
GR: glutathione reductase
MDHAR: monodehydroascorbate reductase
DHAR: dehydroascorbate reductase
AsA: ascorbic acid
DHA: dehydroascorbic acid
GSH: reduced glutathione
GSSG: oxidized glutathione
PGRs: plant growth regulators
FW: fresh weight
DW: dry weight
REC%: relative electric conductivity.
